# Quantifying pollen and spore morphology using a language model

**DOI:** 10.1098/rsbl.2026.0313

**Published:** 2026-10-14

**Authors:** Luke Mander, Peng Sun, Surangi W. Punyasena, Carlos Jaramillo, Francisca Oboh-Ikuenobe

**Affiliations:** ^1^ School of Environment, Earth and Ecosystem Sciences, The Open University, Milton Keynes, UK; ^2^ Institute of Environmental Sciences, Leiden University, Leiden, The Netherlands; ^3^ Department of Plant Biology, University of Illinois, Urbana, IL, USA; ^4^ Smithsonian Tropical Research Institute, Panama City, Panama, Panama City, Panama; ^5^ Department of Earth Sciences and Engineering, Missouri University of Science and Technology, Rolla, MO, USA

**Keywords:** diversity, evolution, taxonomic descriptions, text, digitization

## Abstract

In a typical systematic paper, specimen morphology is described with natural language, and taxonomic specialists use glossaries of technical terminology to establish and communicate taxon concepts, using morphology to delimit species. Textual descriptions of specimen morphology are therefore foundational to biological systematics, but they also serve as a primary data source for studies of morphological evolution. Typically, researchers extract attributes of taxon morphology from descriptions and analyse them to quantify how organismal form has evolved. Focussing on fossil pollen and spores as records of plant evolution, we outline a potential approach to quantifying and comparing morphology that involves using a language model (LM) to transform textual descriptions of specimen morphology into numerical embeddings and then analysing the embeddings statistically. Our results show how an LM can be used to quantitatively analyse the morphological information contained within the text of taxonomic descriptions, illustrating that first-order botanical groupings such as pollen versus spores can be distinguished in the text of a single monograph and potentially from multiple texts by different authors. Although our dataset is small, we tentatively suggest that the spore-plant dominated tropical vegetation of the Carboniferous was comparable in morphological diversity to the angiosperm dominated tropical rainforests of the Palaeogene.

## 1. Introduction

Language models (LMs) provide new opportunities for morphology-based specimen research. Current pioneering applications of LMs in palaeontology use them primarily in specimen classification, querying databases of descriptive text to speed up identifications and standardizing the taxonomic process to improve identification consistency [1,2] (see also unpublished preprint [3]). However, complementary to this, LMs could also be capable of extracting and interpreting the morphological information within the textual descriptions of specimen morphology that are foundational to biological systematics, facilitating the quantification of morphological diversity (used here as a synonym of morphological disparity), and the discovery of evolutionary patterns and phylogenetic relationships from features that are not easily quantified using traditional methods.

In a typical systematic paper, specimen morphology is described with natural language. Taxonomic specialists use glossaries of technical terminology to establish and communicate taxon concepts, using morphological features to delimit species. Specimens of taxa are figured with one or more images, and depending on the field of study, the descriptive text may include some morphometric information such as the size range of the taxon, or the dimensions of certain attributes. This text, in conjunction with figures and first-hand examination of specimens, serves as the primary data source for quantifying morphological evolution. In particular, researchers record attributes such as size [4], the presence or absence of features [5] and discrete character states to describe continuous morphological variation using terms from glossaries [6]. Morphological characters such as these are typically recorded in a matrix, and researchers use measured properties such as the distance between taxa to track the evolution of morphological diversity through time [7–10] (see [11–15] for reviews). In land plants, this approach has shown a pattern of episodically increasing morphological diversity that reflects ecological expansion [16], whereas analyses of extant angiosperm (flowering plant) pollen reveal two major pulses of morphological evolution linked to environmental change and developmental innovations [17]. The largest angiosperm order (Asterales) exhibits a pattern of punctuated bursts of morphological evolution throughout the group's history [18], and fossil angiosperm pollen indicates that the group achieved a high level of morphological diversity early in its evolutionary history [19].

Although the traditional use of morphological characters has provided a wealth of information on the evolution of morphology, the approach is confounded by inapplicable characters [15]. Hierarchical and structurally dependent characters do not apply to certain taxa: characters describing a tail, for example, only apply if a tail exists in the organism. Current methods are unable to process character matrices that include inapplicable characters [15], making them problematic for quantitatively comparing the morphology of phylogenetically distant groups where there is a limited amount of shared homology between groups. Additionally, the use of morphological characters is constrained by the amount of variation that can be described categorically [20].

In contrast to such categorical representations of morphology, LMs provide a means by which the entire text describing the morphology of a taxon can be analysed using natural language processing. Using an LM to extract morphological information contained within text would allow all the observations of the researcher describing a taxon or specimen to be analysed, rather than just those aspects of morphology that are amenable to categorical representation. In the context of pollen and spores, this is an analytical approach that accommodates the possibility that they may be integrated units formed by self-assembly processes during morphogenesis [21–23] and that evolve as a functional whole, rather than representing assemblies of separate morphological characters [24] each evolving independently.

In this paper, we outline a potential approach to quantifying and comparing morphology that involves using an LM to transform textual descriptions of specimen morphology into numerical embeddings and then analysing the embeddings statistically. We are focussed on fossil pollen and spores as records of plant evolution [25], and our primary motivation is to unlock the full potential of the morphological and evolutionary information contained within textual descriptions of specimen morphology. This approach mirrors recent advances in the analysis of pollen images using machine learning methods [26–28]. We apply this approach to fossil pollen and spores from two major vegetation types that dominated tropical latitudes at different periods in Earth's history. The first is the Carboniferous coal forest that grew in the Pennsylvanian (approx. 323–299 Ma) [29] and was composed of giant lycopods and an understory of ferns, seed-ferns and horsetails [30,31]. The second is the Palaeogene tropical rainforest that first emerged following the end-Cretaceous mass extinction (approx. 66 Ma) and was dominated by angiosperms with a closed canopy and multistratal structure [32]. These two time periods represent a challenge to existing character-based approaches to quantifying morphology. Carboniferous coal forests were dominated by plants that produced spores, whereas Palaeogene rainforests were dominated by angiosperms that produce pollen. Because pollen and spores represent different parts of the plant life cycle and have vastly different morphologies [33], inapplicable characters would hamper quantitative comparison of pollen and spore morphologies in the two time periods using morphological characters.

Our main results are twofold. Methodologically, we show how an LM can be used to quantitatively analyse morphological information contained within descriptive text, illustrating that first-order botanical groupings such as pollen and spores can be distinguished in the text of a single monograph (figure 1) and potentially from multiple texts by different authors (figure 2). Although our dataset is small and a larger number of texts is required for a comprehensive macroevolutionary analysis, our results provide a hint that the spore-plant dominated tropical vegetation of the Carboniferous was as complex as the angiosperm dominated tropical rainforests of the Palaeogene, at least as recorded by the morphological diversity of pollen and spores from the Bristol Coalfield (UK; approx. 310 Ma) and the northern Niger Delta (Nigeria; approx. 56 Ma; figure 3). Our approach highlights how descriptive text could be quantitatively analysed in the context of evolutionary investigations of the history of life and potentially integrated with quantitative analyses of specimen-based image data [26–28] generated through large-scale digitization efforts [34].

**Figure 1.**
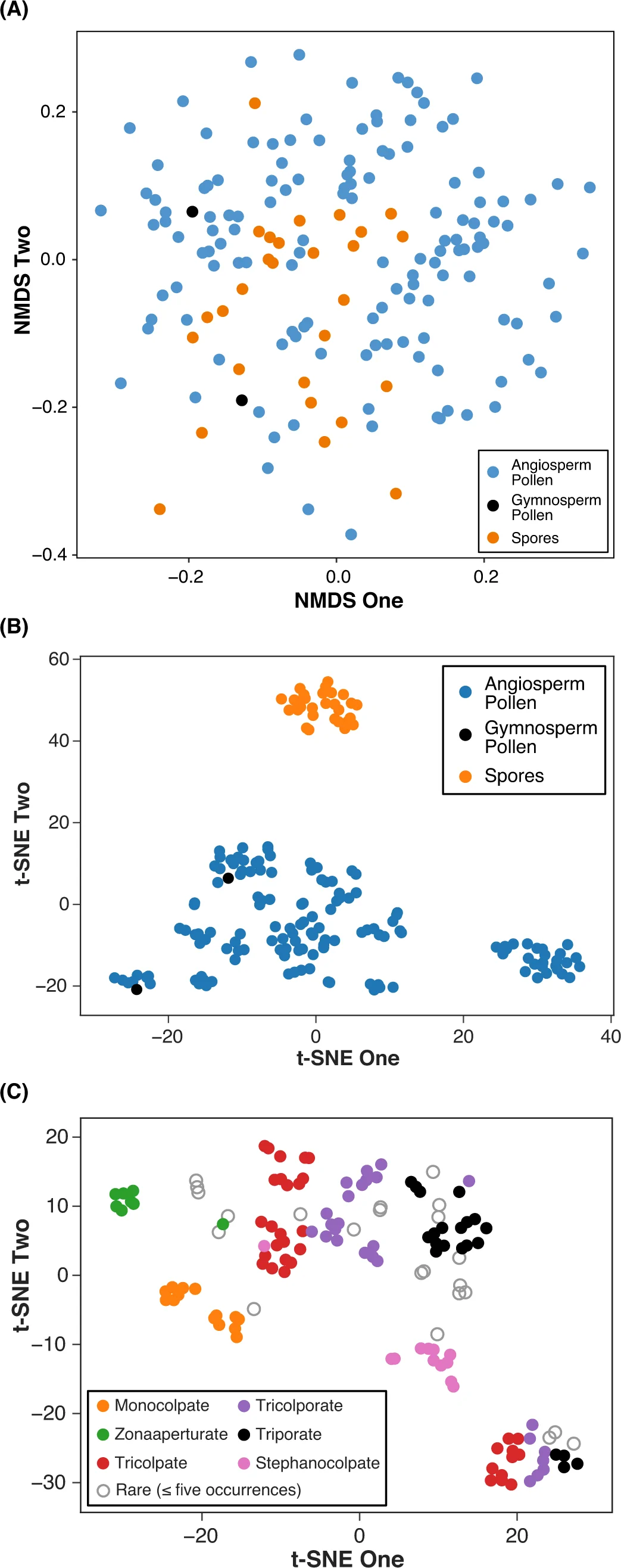
(A) NMDS of morphological characters scored for Palaeogene angiosperm pollen, gymnosperm pollen and spores (stress = 0.265). (B) t-SNE plot of the LM embedding space of Palaeogene angiosperm pollen, gymnosperm pollen and spores (perplexity = 10). (C) t-SNE plot mapping aperture type onto the embedding space of Palaeogene angiosperm pollen (diagnoses included; Perplexity = 10).

**Figure 2.**
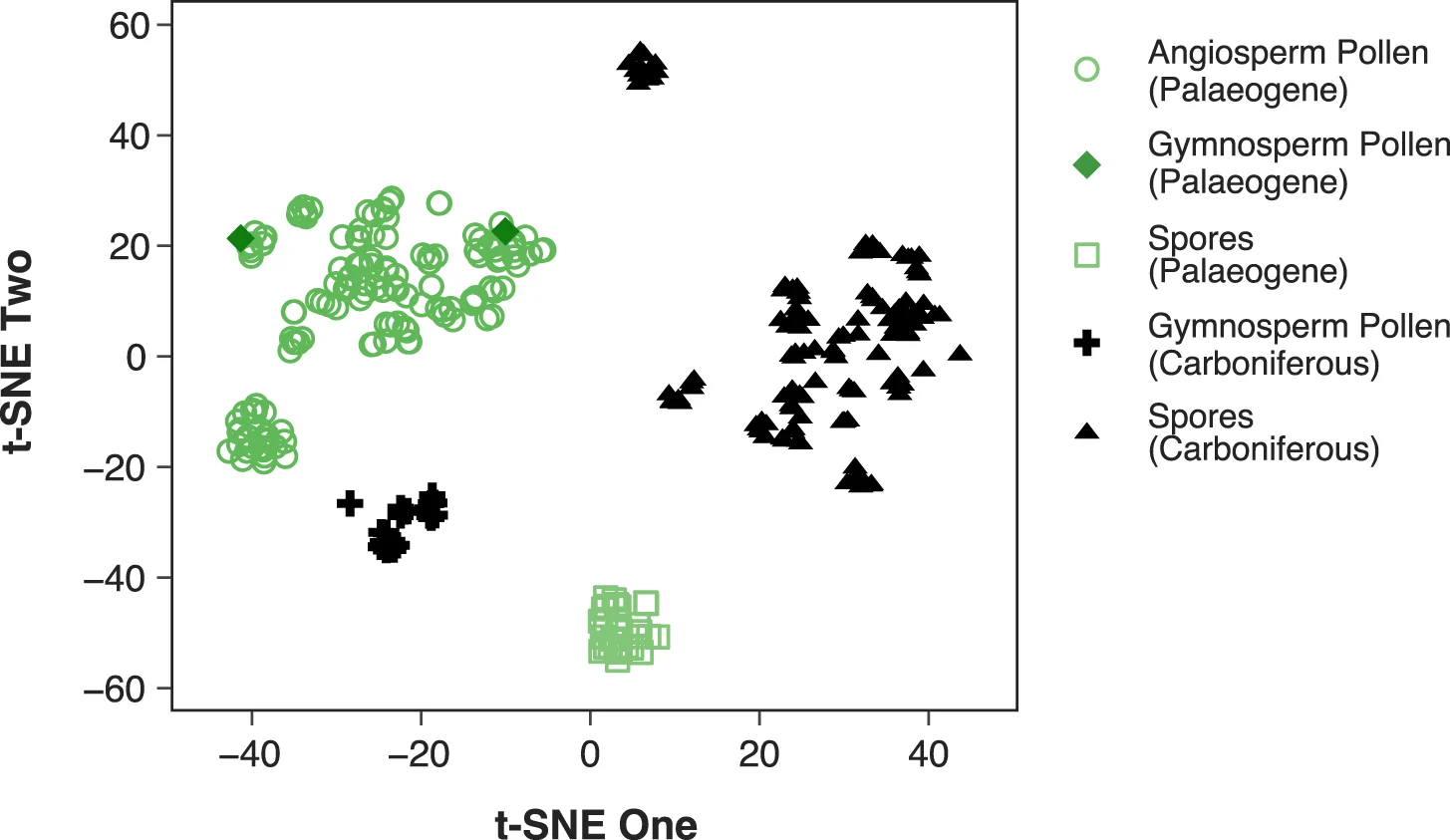
t-SNE plot of the LM embedding space of Palaeogene and Carboniferous pollen and spores (perplexity = 10).

**Figure 3.**
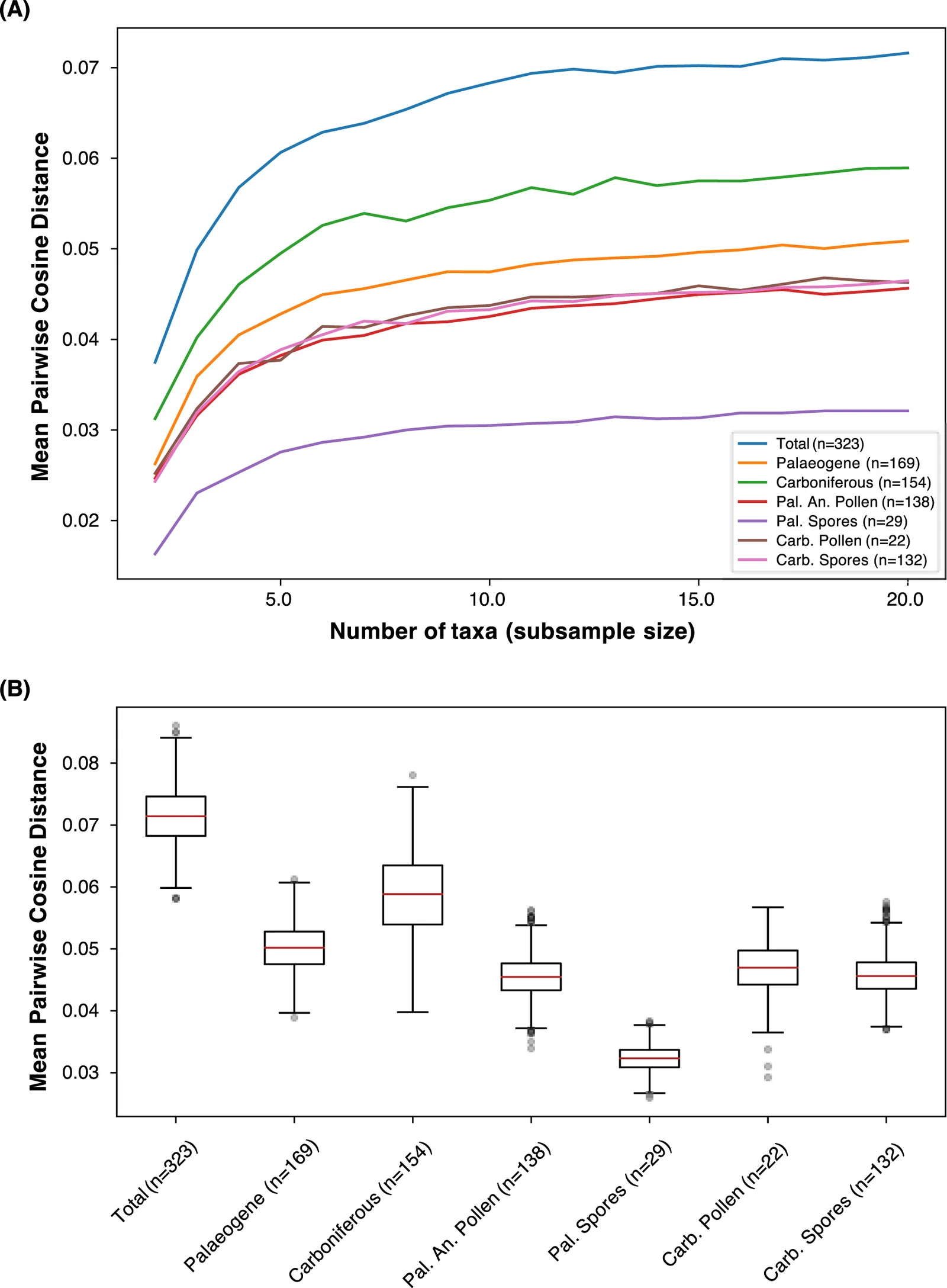
(A) Mean pairwise cosine distance at different subsample sizes. For each subsample size 2, 3, 4, …, 20, taxa were drawn from the group with replacement (200 replicates), and mean pairwise cosine distance was averaged. (B) Boxplot of mean pairwise cosine distances (1000 bootstrap replicates with 20 randomly selected taxa per group).

## 2. Material and methods

### (a) Scoring and analysis of morphological characters

We scored each taxon in [20] for one of 16 morphological characters to generate a matrix (supplementary material). Missing data were scored ‘?’ and inapplicable characters scored ‘NA’. The characters were selected to provide a detailed description of morphological variation without preferentially inflating the number of inapplicable characters in either pollen or spores. Distances between taxa were measured with Gower's similarity metric and the distance matrix was analysed using non-metric multidimensional scaling (NMDS; following [16]; supplementary material).

### (b) Textual descriptions of pollen and spore morphology

Taxonomic descriptions of fossil pollen and spores were compiled from [35] (Carboniferous, Bristol Coalfield; spore-plants and gymnosperms) and [20] (spore-plants, gymnosperms and angiosperms; supplementary material). The descriptions of Carboniferous pollen and spores comprised a total of 154 taxa, with 132 spores and 22 gymnosperm pollen [35]. The descriptions of Palaeogene pollen and spores comprised a total of 169 taxa, with 29 spores, two gymnosperm pollen and 138 angiosperm pollen [20]. Each description uses terminology to report key morphological features that together represent the overall morphology of each taxon. We chose [20] because it is a terminologically consistent descriptive treatment of a full palynological assemblage with which we are familiar (L.M., C.J., F.O.-I. authored the work). We chose [35] because it is both a full palynological assemblage with consistent terminology and an assemblage with a contrasting taxonomic composition to [20]. They are therefore an appropriate test-bed for our approach. We retained the descriptive text of [35] and [20] in its original form without manual editing. We have done this so that we can explore the possibilities and challenges associated with scaling our approach from one to two monographs to the thousands of descriptive works that are available to study.

### (c) Text embedding generation

To enable quantitative comparison of textual morphological descriptions, we first transformed them into fixed-length numerical embeddings. Morphological descriptions were encoded using the multilingual-e5-large-instruct model [36], a transformer-based LM trained to produce dense vector representations that preserve semantic content. The model maps input text, in this case taxonomic descriptions of pollen and spores, into a shared high-dimensional embedding space. The multilingual-e5-large-instruct model was implemented using the SentenceTransformers framework [37] with default parameters. No model training, additional fine-tuning or parameter updating was performed before encoding. All model and inference parameters were kept at their default settings in the SentenceTransformers implementation (supplementary material). We selected this model because at the time of analysis, it ranked within the top 10 models on the multilingual Massive Text Embedding Benchmark [38] leaderboard despite having only approximately 0.5 B parameters. Its modest size therefore means that our work is reproducible on widely available hardware, allowing a low barrier to entry that we regard as important for community adoption of our approach. Descriptions were encoded in batches of 32 using NVIDIA GTX 3090 GPU acceleration. Each taxon description was converted into a single 1024-dimensional embedding vector derived from pre-trained model weights (supplementary material). Descriptions in [35] lacked a diagnosis section, and this material was removed from the descriptive text of [20] before embedding to facilitate comparison between the two datasets. The full descriptive text (including the diagnosis) was included in an embedding of angiosperm pollen descriptions to facilitate an analysis of aperture type.

### (d) Analysis of numerical embeddings

We measured distances between taxa using cosine similarity, the cosine of the angle between two embedding vectors, which we use as a disparity metric. We used t-distributed stochastic neighbour embedding (t-SNE) to visualize the distribution of pollen and spores in the 1024-dimensional embedding space (supplementary material). All distances and statistical tests were computed in the full embedding space, with t-SNE and Uniform Manifold Approximation and Projection (UMAP) used only for visualization.

## 3. Results

NMDS analysis of morphological characters fails to resolve Palaeogene pollen versus spores into distinct clusters (figure 1A). By contrast, our LM approach clearly distinguishes pollen from spores in this dataset (figure 1B). An analysis of aperture type in Palaeogene angiosperm pollen shows several clusters dominated by different aperture types, with rare apertures spread across embedding space (figure 1C). A cluster of taxa with high t-SNE one and negative t-SNE two scores in the lower right of the plot is composed of taxa with tricolpate, tricolporate and triporate apertures, with three taxa that have rare aperture types (figure 1C). Our approach also partitions Carboniferous and Palaeogene pollen and spores into groups (figure 2), and this is achieved with no training objectives. Visualization using UMAP recovers the same underlying groupings (supplementary material). Comparison of pairwise cosine distance among different groups indicates that Carboniferous pollen and spores as a whole group are more diverse morphologically than Palaeogene pollen and spores (Mann–Whitney *U*-test; *p* < 0.0001), whereas Palaeogene spores have the lowest morphological diversity (Mann–Whitney *U*-test; *p* < 0.0001; figure 3). Carboniferous spores and Palaeogene angiosperm pollen dominate the two datasets (*n* = 132 and *n* = 128, respectively) and are very similar in their morphological diversity, with Carboniferous spores marginally higher (Mann–Whitney *U*-test; *p* < 0.0087; figure 3).

## 4. Discussion

The multilingual-e5-large-instruct model was able to effectively differentiate between pollen and spores in the Palaeogene (figure 1B) and the Carboniferous (figure 2), and between pollen and spores of the two time periods (figure 2) based on textual descriptions alone. This demonstrates the ability of LMs and vector representations of descriptive text to uncover evolutionary patterns contained within morphological accounts produced by taxonomists. Our approach adds a new dimension to the quantification and analysis of morphology, complementing recent advances in the quantitative analysis of biological imagery [26–28] and opening a new window into macroevolutionary analyses of form.

Part of the motivation for using LMs to quantify morphology is to overcome the barriers presented by morphological characters and the use of traditional character matrices. Previous attempts to overcome these barriers have included using distance metrics such as the Gower index that can handle missing data [16], but this does not resolve the issues presented by inapplicable characters [15] (figure 1A). In studies of plant vegetative structures [16], there may be sufficient overlap in morphological characters to facilitate comparison, but this is not the case for spores and pollen, which are characterized by distinctively primitive and derived characters. Workers have also developed measures of morphology that are sufficiently general to allow morphological complexity to be quantified [39], but these have been developed for plant reproductive structures (cones and flowers) that are differentiated into parts that are amenable to discretization and counting [40]. By contrast, although pollen and spores can be viewed as collections of discrete characters that each can be described [6] and analysed individually [24], certain morphological features can form by self-assembly processes [21–23]. This raises the possibility that pollen and spores could evolve as a whole integrated unit, which to a degree questions the use of morphological characters as appropriate descriptors. However, even if the morphological characters that make up a pollen grain or spore do each evolve independently, current character-based methods are not well-suited to the quantification of pollen and spore morphology (figure 1A), further emphasizing the need for new methods such as the LM approach we describe here.

The separation of pollen from spores within the Palaeogene (figure 1B) and within the Carboniferous (figure 2) indicates that the model recovers morphological categories rather than a simple dataset-of-origin signal. Two Palaeogene gymnosperm pollen grains do not form a discrete cluster of their own but overlap with angiosperm pollen from the same interval (figure 1B). This is probably because these two gymnosperm pollen grains are simple forms with few distinguishing features, with *Cycadopites* a smooth monocolpate form and *Cyclusphaera* a simple diporate form (see [6] for terminology), which are both morphological types found commonly among angiosperm pollen. This further demonstrates that the embeddings recover primarily morphological categories as recorded by text. Additionally, for angiosperm pollen, the distribution of taxa in embedding space is partly structured by aperture type (figure 1C), which is expected because apertures are prominent aspects of descriptions of angiosperm pollen morphology. However, the embeddings do not group taxa only by a single feature such as apertures: the cluster to the lower right of figure 1C shows that other aspects of the descriptions also contribute to the separation and clustering of taxa.

The separation between the Carboniferous and Palaeogene datasets (figure 2) is more challenging to interpret. First, the two could potentially be distinguished by the compositional semantics of different workers ([35], Carboniferous and [20], Palaeogene). However, general-purpose text embedding models are optimized for semantic similarity rather than stylistic attributes. Standard embeddings preferentially encode content over writing style, and isolating authorship signal from content requires specialized contrastive training objectives not used here [41,42]. This bias towards content is reinforced in our case by the terminological framework of palynology, which constrains the lexical space available for morphological descriptions and means that the primary signal accessible to the model is scientific rather than idiolectal.

Second, there are variations in the terminology used by different workers. In this case, the descriptions of Mander *et al.* [20] strictly and exclusively used the terminology listed in [6], whereas the descriptions of Pendleton [35] used terminology from [43] that reflected earlier works [44–47], with the terms of [6] also used. Specific terminological differences in the two texts are as follows. For smooth surface ornamentation, Pendleton uses *laevigate*, but Mander *et al.* use *psilate*. For the exine, Mander *et al.* use the term *exospore* for the spore wall and *exine* for the pollen wall, whereas Pendleton uses the term *exine* for the wall of both spores and pollen. Unlike Mander *et al.* [20], Pendleton [35] does not always measure exine thickness and does not discriminate between one- and two-layered spore exines. Unlike Mander *et al.* [20], Pendleton [35] does not use the terms *monad* or *tetrad* to describe the dispersal unit because all spores and pollen in the Carboniferous dataset are dispersed as monads (for the same reason, the term *polyad* does not appear in [20] because there were no polyads in the Palaeogene dataset).

Despite these differences, the descriptions in the two texts share a majority of descriptive terms. For example, for spores, both texts use the term *amb* to describe the spore outline, use descriptors such as *triangular* to report the shape of the amb, and use the term *laesurae* to describe the nature of the *trilete* mark, which partitions spore taxa into *alete*, *monolete* and *trilete* groups. Both texts therefore use the same terms to describe the fundamental building blocks of the pollen and spore bauplan. Consequently, we interpret the separation of the Carboniferous and Palaeogene pollen and spores (figure 2) as primarily reflecting morphological differences, while acknowledging that some of the separation is probably due to the same feature being described with a different term in each text (e.g. *laevigate* vs. *psilate*). This crystalizes a key issue with applying our approach to a greater number of texts: how to contend with the different terminology used by different workers? Descriptions of pollen and spores produced today use different terms compared to those produced by early workers [48], and given the large and well-developed literature of descriptive palynology in English, French, German, Spanish and Russian [49], further work in this area will also need to confront different languages. Although these terminological and linguistic differences are a major challenge, contextual embeddings may partly address them. Because word meaning is inferred from surrounding context [50], synonymous terms used in similar morphological contexts typically plot closely together in embedding space, while multilingual models such as the model we have used here [36] may also help bridge language barriers. Nevertheless, differences in text structure, authorial style and historical idiom may still introduce non-morphological signals, which future work could reduce through structured entity extraction or light-touch rule-based rewriting before embedding. Task-specific fine-tuning would also be a sensible next step for taxonomic identification applications [3].

Our analysis of Carboniferous and Palaeogene pollen and spores is based on descriptions of palynomorphs from two localities that represent a subset of the entire flora for each time period. Nevertheless, our results (figure 3) cast doubt on current views of the relative morphological diversity of these two time intervals. In particular, analyses of fossil reproductive structures (e.g. cones and flowers) suggest that the evolution of angiosperms led to a marked rise in morphological complexity linked to the increased number of functions that a flower has to perform compared to a sporangium [38], but our analyses provide a hint that this pattern may not be replicated by pollen and spores. The spores from the tropical Carboniferous forests [35] are at least as diverse as the angiosperm pollen from the tropical Palaeogene rainforest samples [20]. We hypothesize that the larger, or at least comparable, levels of morphological diversity among spores from Carboniferous tropical coal forests (figure 3) may relate to spore dispersal, which probably involved diverse biotic and abiotic mechanisms [51]. However, we emphasize that this represents a provisional inference and our data are currently too limited to draw any definitive macroevolutionary conclusions. Analyses of more primary descriptive works at the species level in both regions, which also account for differences in regional geological setting, are required to fully test this hypothesis.

Finally, both images and textual descriptions of biological specimens can be encoded as vector representations, and consequently there is the potential to align these two modalities within a shared embedding space (e.g. Contrastive Language–Image Pre-training [52]), leveraging the morphological knowledge embedded in taxonomic descriptions to guide visual models towards finer-grained taxonomic classification and the inference of additional morphological attributes. Such an approach would use a massive but underused resource: the multitude of pollen and spore descriptions of both extant and fossil taxa from the past century that are buried in hundreds of catalogues and thousands of descriptive monographs. This would build on recent advances in the quantification of pollen and spore morphology using a combination of high-resolution microscopy, computational image analysis and machine learning techniques, which has improved the taxonomic resolution of classifications [28,53,54] and allowed for specimens of fossil pollen to be placed within molecular phylogenies [26,27], and is complementary to chemotaxonomic methods that have enhanced the taxonomic and palaeoecological information extracted from fossil pollen and spores [55–57]. It could also enrich the results of large-scale digitization of pollen and spore collections [34] and natural history collections more broadly.

## Supplementary Material



## Data Availability

All data and code have been included in the supplementary material. Supplementary material is available online [58].
