# Supplementary material for: Quantifying pollen and spore morphology using a language model
Source: Biol Lett. 2026 Oct 14;22(10):20260313. doi: 10.1098/rsbl.2026.0313 (PMC13682553; doi:10.1098/rsbl.2026.0313)
Supplement: Supplementary file 1 [file rsbl.2026.0313.zip › rsbl.2026.0313/rsbl20260313_si_001.pdf]

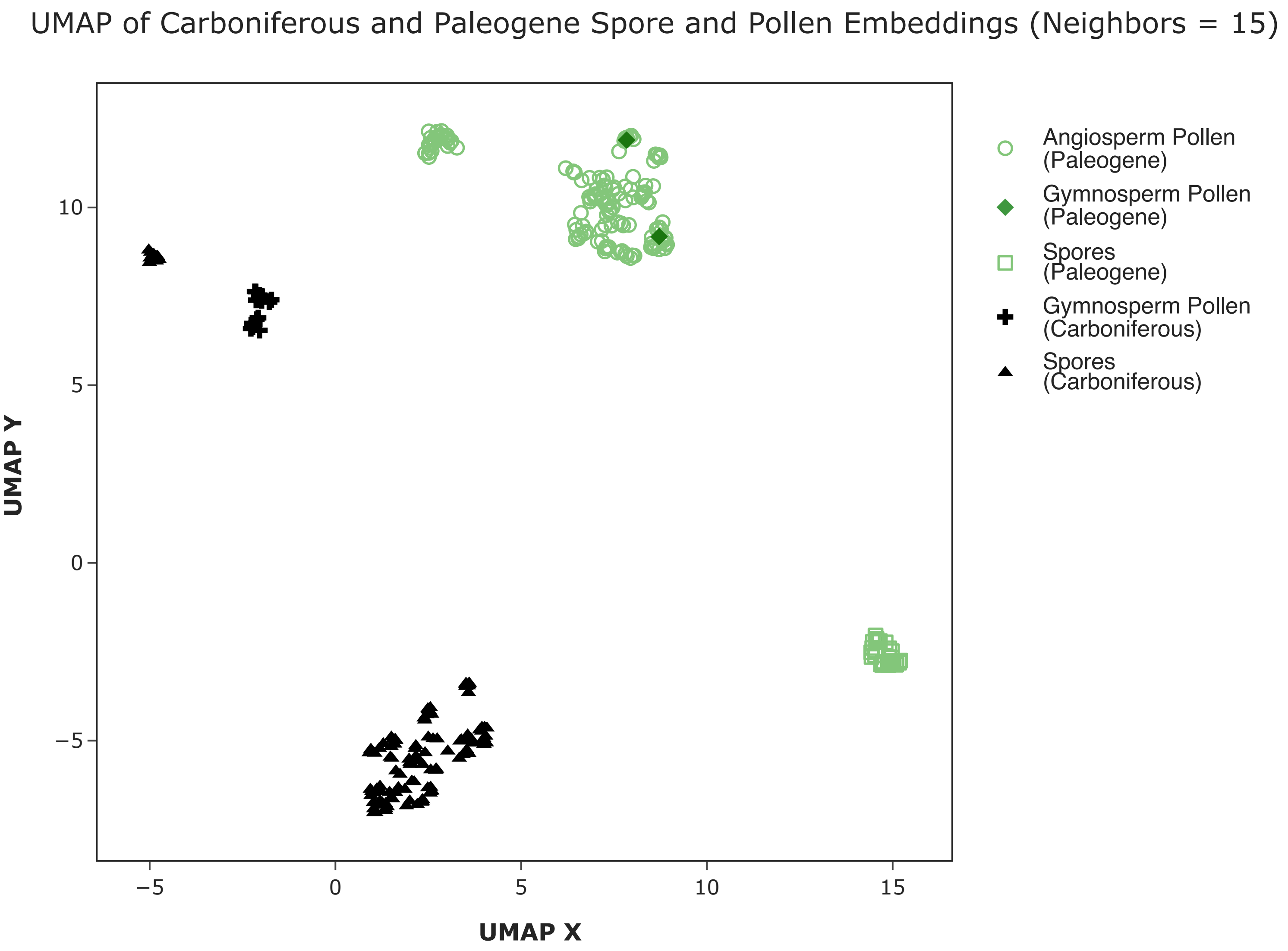
UMAP of Carboniferous and Paleogene Spore and Pollen Embeddings (Neighbors = 15)
UMAP Y
UMAP X
10
5
0
−5
−5
0
5
10
15
Angiosperm Pollen (Paleogene)
Gymnosperm Pollen (Paleogene)
Spores (Paleogene)
Gymnosperm Pollen (Carboniferous)
Spores (Carboniferous)

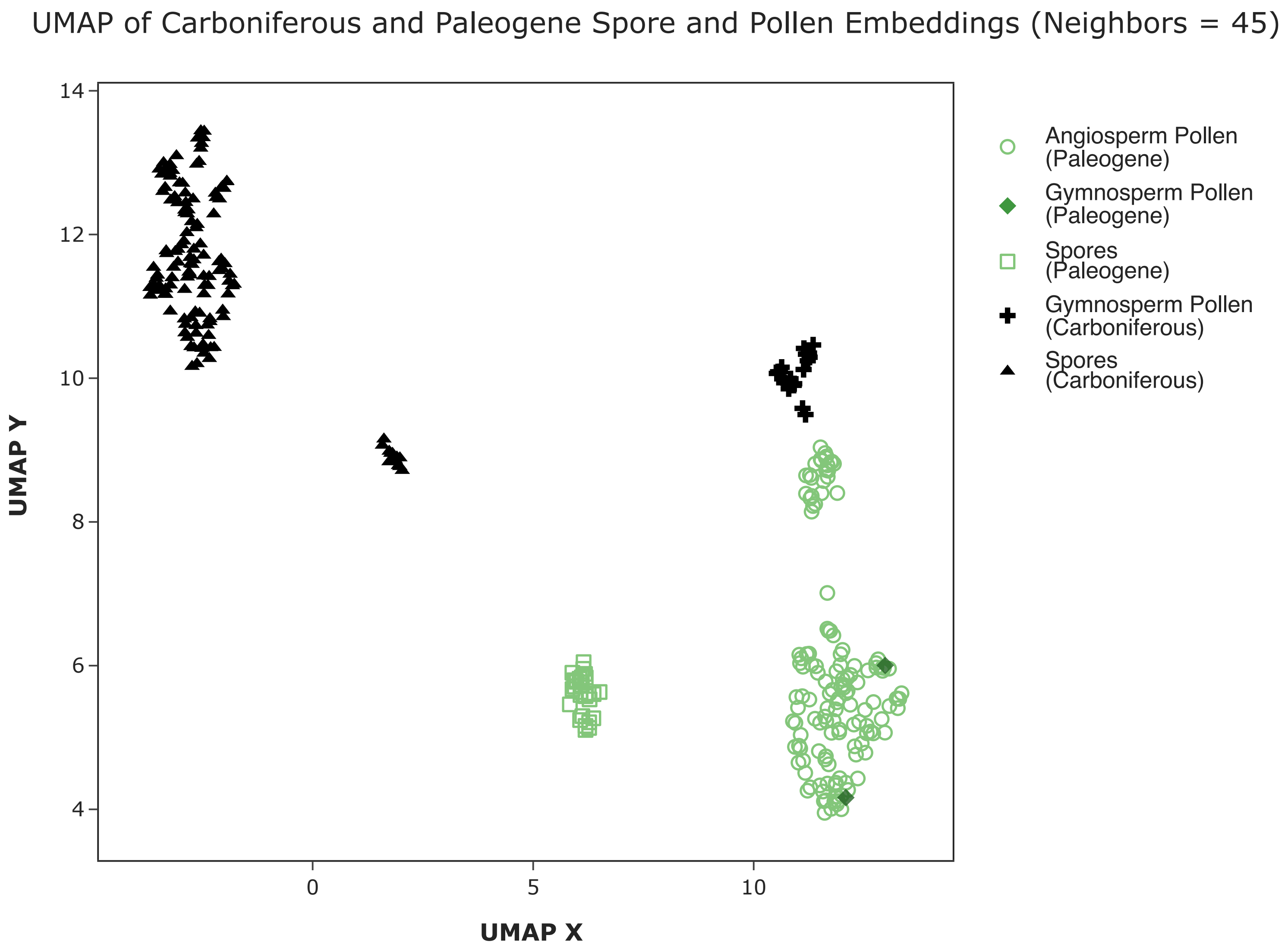
UMAP of Carboniferous and Paleogene Spore and Pollen Embeddings (Neighbors = 45)
UMAP Y
UMAP X
14
12
10
8
6
4
0
5
10
Angiosperm Pollen (Paleogene)
Gymnosperm Pollen (Paleogene)
Spores (Paleogene)
Gymnosperm Pollen (Carboniferous)
Spores (Carboniferous)
