# Supplementary material for: Quantifying pollen and spore morphology using a language model
Source: Biol Lett. 2026 Oct 14;22(10):20260313. doi: 10.1098/rsbl.2026.0313 (PMC13682553; doi:10.1098/rsbl.2026.0313)
Supplement: Supplementary file 1 [file rsbl.2026.0313.zip › rsbl.2026.0313/rsbl20260313_si_002.docx]

**Morphological Characters and Character States**

**1. Dispersal unit**

0 = monad; 1 = tetrad

**2. Shape**

0 = circular; 1 = sub-circular; 2 = elliptic; 3 = oval; 4 = reniform; 5 = triangular; 6 = sub-triangular; 7 = oblate; 8 = sub-oblate; 9 = prolate; 10 = sub-prolate; 11 = angular/polygonal

**3. Exine stratification**

0 = atectate; 1 = intectate; 2 = semitectate; 3 = tectate; NA if spore

**4. Tectal columellae**

0 = indistinct; 1 = distinct; N/A if exine stratification 0, 1 or NA.

**5. Exine thickness**

0 = <0.5µm; 1 = 0.5–1µm; 2 = 1–1.5µm; 3 = 1.5–2µm; 4 = >2µm

**6. Tetrad mark**

0 = alete; 1 = monolete; 2 = trilete; N/A if angiosperm pollen or gymnosperm pollen lacking a tetrad mark.

**7. Tetrad mark variation (e.g. labrum, kyrtome)**

0 = absent; 1 = present; N/A if alete or if angiosperm pollen or gymnosperm pollen lacking a tetrad mark.

**8. Valvae**

0 = absent; 1 = present; N/A if angiosperm pollen or gymnosperm pollen.

**9. Cingulum**

0 = absent; 1 = present; N/A if angiosperm pollen or gymnosperm pollen.

**10. Aperture**

0 = Inaperturate; 1= Monoporate; 2 = Diporate; 3 = Triporate; 4 = Pantoporate; 5 = Stephanoporate; 6 = Monocolp(sulc)ate; 7 = Dicolpate; 8 = Tricolpate; 9 = Stephanocolpate; 10 = Syncolpate; 11 = Parasyncolpate; 12 = Trichotomocolp(sulc)ate; 13 = Heterocolpate; 14 = Monocolporate; 15 = Dicolporate; 16 = Tricolporate; 17 = Stephanocolporate; 18 = Syncolporate; 19 = Parasyncolporate; 20 = Zonasulcate; 21 = Tetracolpate; 22 = Tetracolporate; N/A if Tetrad mark = 0, 1 or 2.

**11. Aperture variation (e.g. Annulus, Margo, Costa**, **Operculum. Vestibulum/Fastigium)**

0 = absent; 1 = present; N/A if Inaperturate or if Tetrad mark = 0, 1 or 2

**12. Primary surface ornamentation**

0 = Psilate; 1 = Scabrate; 2 = Granulate; 3 = Baculate; 4 = Echinate; 5 = Microechinate; 6 = Gemmate; 7 = Microgemmate; 8 = Verrucate; 9 = Microverrucate; 10 = Clavate; 11 = Pilate; 12 = Rugulate; 13 = Striate; 14 = Reticulate homobrochate; 15 = Reticulate heterobrochate; 16 = Foveolate; 17 = Micropitted; 18 = Perforate; 19 = Fossulate; 20 = Areolate; 21 = Plicate

**13. Secondary surface ornamentation**

0 = Psilate; 1 = Scabrate; 2 = Granulate; 3 = Baculate; 4 = Echinate; 5 = Microechinate; 6 = Gemmate; 7 = Microgemmate; 8 = Verrucate; 9 = Microverrucate; 10 = Clavate; 11 = Pilate; 12 = Rugulate; 13 = Striate; 14 = Reticulate homobrochate; 15 = Reticulate heterobrochate; 16 = Foveolate; 17 = Micropitted; 18 = Perforate; 19 = Fossulate; 20 = Areolate; 21 = Plicate;

22 = None

**14. Variation in sculptural elements**

0 = no systematic variation; 1 = sculptural elements vary systematically in density and/or size and/or arrangement across exine (e.g. *Rousea*); N/A if surface ornamentation psilate.

**15. Ornamentation on proximal versus distal face**

0 = the same; 1 = different; N/A if angiosperm or gymnosperm pollen.

**16. Size**

0 = <15µm; 1 = 15–30µm; 2 = 30–45µm; 3 = 45–60µm; 4 = 60–75µm; 5 = 75–90µm; 6 = >90µm
